# Impairment on Cardiopulmonary Function after Marathon: Role of Exhaled Nitric Oxide

**DOI:** 10.1155/2019/5134360

**Published:** 2019-02-17

**Authors:** Ana Paula Sierra, Manoel Carneiro Oliveira-Junior, Francine Maria Almeida, Marino Benetti, Rodrigo Oliveira, Soraia Nogueira Felix, Isabella Santos Genaro, Beatriz Mangueira Saraiva Romanholo, Nabil Ghorayeb, Maria Augusta Peduti Dal Molin Kiss, Maria Fernanda Cury-Boaventura, João Bosco Pesquero, Rodolfo Paula Vieira

**Affiliations:** ^1^School of Physical Education and Sport, University of São Paulo, São Paulo, Brazil; ^2^Sports Cardiology Department, Dante Pazzanese Institute of Cardiology, São Paulo, Brazil; ^3^Nove de Julho University, São Paulo, Brazil; ^4^Laboratory of Experimental Therapeutic (LIM 20), School of Medicine, University of São Paulo, São Paulo, Brazil; ^5^Institute of Physical Activity and Sports Sciences (ICAFE), Cruzeiro do Sul University (UNICSUL), São Paulo, Brazil; ^6^Department of Biophysics, Federal University of Sao Paulo (UNIFESP), São Paulo, Brazil; ^7^Post Graduation Program in Bioengenering and in Biomedical Engineering, Universidade Brasil, São Paulo, Brazil; ^8^Post Graduation Program in Sciences of Human Movement and Rehabilitation, Federal University of São Paulo (UNIFESP), Santos, Brazil; ^9^Brazilian Institute of Teaching and Research in Pulmonary and Exercise Immunology (IBEPIPE), São José dos Campos, Brazil; ^10^School of Medicine, Anhembi Morumbi University, São José dos Campos, Brazil

## Abstract

**Background:**

The endurance exercise is capable of inducing skeletal muscle, heart, and respiratory fatigue, evidenced by morphofunctional cardiac changes, release of myocardial injury biomarkers, and reduction of maximal voluntary ventilation and oxygen consumption (VO_2_) at peak exercise.

**Purpose:**

The aim of this study was to investigate whether marathoners present cardiac fatigue after marathon and whether it correlates with pulmonary levels of exhaled nitric oxide (eNO) and pulmonary inflammation.

**Methods:**

31 male marathoners, age 39 ± 9 years, were evaluated by cardiopulmonary exercise test three weeks before and between three and 15 days after a marathon; eNO analysis and spirometry were evaluated before, immediately after, and 24 and 72 hours after the marathon, and sputum cellularity and cytokine level were assessed before and after the marathon.

**Results:**

Marathon induced an increase in the percentage of macrophages, neutrophils (from 0.65% to 4.28% and 6.79% to 14.11%, respectively), and epithelial cells and a decrease in cytokines in induced sputum, followed by an increase in eNO concentration (20 ± 11 to 35 ± 19 ppb), which presented a significant reduction 24 and 72 hours after marathon (9 ± 12 e 12 ± 9 ppb, *p* < 0.05). We observed a decrease in the spirometry parameters in all time points assessed after the marathon (*p* < 0.05) as well as in cardiopulmonary capacity, evidenced by a reduction in VO_2_ and ventilation peaks (57 ± 6 to 55 ± 6 mL·min^−1^·Kg^−1^ and 134 ± 19 to 132 ± 18 Lpm, respectively, *p* < 0.05). Finally, we observed a negative correlation between the decrease in forced expiratory volume and decrease in eNO 24 and 72 hours after marathon (*r* = −0.4, *p* = 0.05).

**Conclusion:**

Reduction in eNO bioavailability after marathon prevents the reduction in cardiopulmonary capacity induced by acute inflammatory pattern after marathon.

## 1. Introduction

Previous studies indicated the oxygen transport to the muscle as a major factor to endurance exercise performance. Impairment of this process reduces VO_2max_, compromises endurance performance, and can promote the fatigue development and decrease in cardiopulmonary function [1]. The “oxygen hypothesis” claims that after prolonged strenuous exercise, there is a reduction in oxygen gas exchange and reduction in oxygen perfusion capacity in the skeletal muscle periphery due to a reduced diffusion capacity [2].

The exhaled nitric oxide (eNO) is centrally involved in the regulation of pulmonary gas exchange [3] and in oxygen perfusion in muscle periphery [4]. In fact, a study has demonstrated that reduced levels of eNO correlates with increased exercise intolerance and with impairment on ventilatory response [3]. eNO is considered a valuable marker of airway inflammation, especially in respiratory diseases, such as asthma [5], chronic obstructive pulmonary disease [6, 7], acute respiratory distress syndrome [8], and in idiopathic pulmonary fibrosis [5–9].

Air pollution also may influence airway inflammation response during exercise [10]. Previous studies suggest that particulate matter induces oxidative and nitrosative stress as a result of superoxide and nitric oxide reaction, which downregulate NO synthase and reduces NO availability, consequently leading to vasoconstriction [11, 12].

The level of eNO has been reported to associate with bronchoconstriction and reduction on pulmonary function, especially in forced expiratory volume in 1 second (FEV1) during exercise, as a result of inflammation and flow limitations [13–15].

Therefore, the present study was aimed at investigating whether marathon causes cardiac fatigue and, if it is the case, whether cardiac fatigue correlates with pulmonary levels of eNO and pulmonary inflammation.

## 2. Methods

### 2.1. Subjects

Thirty-one male marathoners (age 39 ± 9 years) participated in the XXI São Paulo International Marathon, held on May 17th 2015. All individuals were aware of the possible risks involved in the study and provided a written informed consent. The study conformed to the declaration of Helsinki and the protocol was approved in advance by the institutional ethics committee of Dante Pazzanese Institute of Cardiology and of School of Physical Education and Sport. The exclusion criteria included the use of medication to cardiac, metabolic, pulmonary, or kidney injury, use of alcohol or any kind of drugs, and pathologies including systemic arterial hypertension and liver, kidney, metabolic, inflammatory, or neoplasic diseases.

### 2.2. Cardiopulmonary Test

The cardiopulmonary tests were performed from three weeks to two days before and from three days to fifteen days after marathon using a ramp protocol. The protocol began with 8 km/h and 1% fixed slope, increasing 1 km/h per minute until maximal exhaustion of the marathoner. Expired gas analysis was performed in a breath-by-breath system (Ergostik®, Geratherm, Bad Kissingen, Germany). Room conditions were controlled and temperature was maintained between 21°C and 23°C. Air humidity was registered by a system (Ambistik®, Bad Kissingen, Germany). System was calibrated manually using a 3-liter syringe in the beginning of the section and every two tests.

### 2.3. Spirometry

Pulmonary function was measured using a *Spirostik*®(Geratherm, Bad Kissingen, Germany) spirometer by a trained technician 24 h before, immediately after, and 24 hours and 72 hours after marathon to obtain the parameters forced vital capacity (FVC), forced expiratory volume in one second (FEV1), the relation FEV1/FVC, and peak of expiratory flow (PEF), considering the criteria for acceptability and reproducibility, according to the recommendations of European Respiratory Society and the guidelines for pulmonary function test.

### 2.4. Nitric Oxide in Exhaled Air

Nitric oxide in exhaled air was measured 24 h before, immediately after, and 24 hours and 72 hours after marathon. For this procedure, we used a flow valve, an attached nozzle, and a 1.5-litter Mylar bag. The marathoners performed a controlled expiration of 12 liters per minute (controlled by the values shown in the flow valve) for eight seconds, after which the Mylar bag was quickly closed. After the procedure, the flask was emptied in the nitric oxide analyser (NO Analyzer, GE, Sievers), which uses the chemiluminescence technique to measure nitric oxide in exhaled air. System was calibrated with a cylinder with known nitric oxide concentration.

### 2.5. Cellularity and Cytokines in Induced Sputum

Sputum was induced by inhalation of hypertonic saline aerosol. Marathoners were instructed to blow their noses and rinse their mouths out with water, inhale nebulized 3% saline for 10 minutes, and then instructed to expectorate their sputum into sterile pots. Sputum was separated from contaminants and saliva, placed in a centrifugal tube, and centrifuged for 10 min at 1200 g at 4°C. The supernatant was collected and stored at -80°C. Total cell numbers was determined by cell counting using a hemocytometer (Neubauer chamber) and differential cell counting by cytospin preparations stained with Diff-Quik staining, where 300 cells were analysed and counted according to the haematological and morphological criteria in a blinded fashion for individual and time point description.

The concentrations of cytokines were measured using ELISA kits (DuoSet, R&D Systems, MN USA), according to the manufacturer's instructions.

### 2.6. Statistical Analysis

Statistical analysis was performed in SPSS software 22.0 (IBM Inc., Chicago IL, 2013). Kolmogorov-Smirnov test was performed and the data rejected normality in most variables. To analyse the marathon's impact on data during the four steps, we performed Friedman test and Müller-Dunn post test for variables that have more than two moments. For the variables with two moments, we performed Wilcoxon test. Spearman correlation test was applied to identify the correlation between the variables. The results are shown in mean values and standard deviation. The significance level was set to 5%.

## 3. Results

Thirty-one marathoners completed the São Paulo International Marathon and all of the steps of this study. The marathon took place in fall and day was sunny throughout the marathon (temperature between 16.1°C and 22.6°C and humidity between 89% and 61%). Demographic characteristics and marathon time are shown in Table 1.

### 3.1. Increased Airway Inflammation after Marathon

Immediately after the marathon race, the number of macrophages, neutrophils, and epithelial and total cells increased in sputum by 2.5-fold, 4.7-fold, 1.8-fold, and 1.8-fold, respectively (*p* < 0.01). Macrophage percentage changed from 0.65% to 4.28% and neutrophil percentage from 6.79% to 14.11%. Lymphocyte percentage did not show significant changes after marathon (Table 2). In addition, marathon race reduced IL-12p40 (*p* < 0.01), IL-23 (*p* < 0.03), IL-33 (*p* < 0.01), and thymic stromal lymphopoietin (TSLP) levels (*p* < 0.05). However, IL-6 and IL-8 did not show significant changes after marathon compared with basal levels (Table 2).

### 3.2. Changes in Exhaled Nitric Oxide (eNO) after Marathon

**Figure 1:**
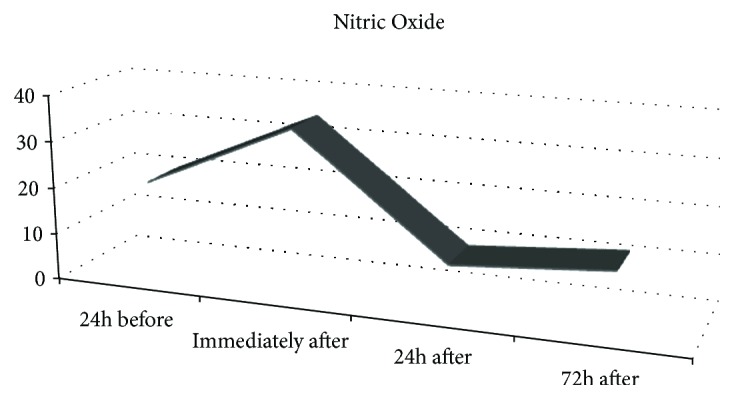
Exhaled nitric oxide before, immediately after, and 24 hours and 72 hours after marathon (ppb).

Exhaled NO increased immediately after the marathon and reduced 24 hours and 72 hours after the race (Figure 1).

### 3.3. Decreased Cardiopulmonary Function after Marathon

Values for FVC, FEV1, and PEF were decreased after marathon and did not return to baseline levels, suggesting an impairment of pulmonary function until three days after the race (Table 3).

The cardiopulmonary function presented a significant decrease in a period after marathon (three to fifteen days after marathon) especially concerning VO_2_ peak, ventilation peak, and PetO_2_ peak, even though the marathoners have reached the same speed in both tests, as shown in Table 4.

We observed a positive correlation between the amount of eNO and the number of macrophages in sputum immediately after marathon (*r* = 0.573, *p* < 0.01) suggesting pulmonary inflammation. Moreover, a negative correlation between the differences in FEV1 72 hours with the difference in nitric oxide values 24 and 72 hours after marathon (*r* = −0.4, *p* < 0.05) was found. Finally, we found a positive correlation between FVC and peak VO_2_ values after marathon (*r* = 0.421, *p* < 0.05).

## 4. Discussion

The major finding of this study was the impairment of pulmonary function and VO_2peak_, associated to airway inflammation, marked by increased levels of exhaled nitric oxide and inflammatory cell number in the sputum of marathoners after a marathon. The bioavailability of exhaled nitric oxide was negatively associated to pulmonary function and with cardiac fatigue after marathon.

Our study showed an increase in epithelial cell number in induced sputum after marathon, suggesting airway injury characterized by epithelial desquamation. Airway epithelial cells protect the airways against external agents, not only by acting as a physical and functional barrier but also by modulating airway inflammation and repair processes [16, 17].

Airway inflammation in athletes has been poorly described and with controversial results in studies focusing this subject [18, 19]. Bonsignore et al. found increases in lymphocytes and eosinophils and a decrease in macrophages in induced sputum after a 5 km race at the beach [20]. Conversely, and in accordance with our study, Larsson et al. observed an increase in inflammatory cells expressed by neutrophils and macrophages in subjects which performed one hour of exercise on a treadmill [21].

Chimenti et al. suggest that increases in inflammatory cells in the airways do not necessarily imply the occurrence of proinflammatory activation because the markers of inflammation in induced sputum were not increased [17]. In addition, we suggest that reduced levels of important airway cytokines impair the pulmonary immune function preventing the resolution of inflammation. IL-12 is a cytokine important in host immunity, produced by activated macrophages, neutrophils, and dendritic cells. The p40 subunit of IL-12 is shared by IL-23, promoting the differentiation of Th1 and Th17 cells, respectively. A previous study suggested that IL-12p40 may be indispensable for downregulation of airway fibrosis and inflammation [22]. In addition, the downregulation of IL12p40 is associated with Th2 induction [23]. However, downregulation of IL-33 and thymic stromal lymphopoietin (TSLP) reduces Th2 differentiation, suggesting an imbalance on airway immune response. These cytokines are proallergenic indicating also low allergenic response as well as impaired airway immune function after marathon running [24]. On the other side, it has been demonstrated that low and moderate intensity aerobic exercise can result in opposite effects in comparison with marathon running, restoring airway immunological balance, although epithelial desquamation persists [25].

In our study, we found increase in eNO immediately after marathon with significant decrease 24 and 72 hours after marathon, with a reduction in bioavailability of eNO in this period. Accordingly, Carbonnelle et al. [26] and De Gouw et al. [27] found an increase in eNO levels after two sessions of swimming and after cycling, respectively. A direct relationship between acute exercise intensity and the levels of eNO in sedentary healthy and trained subjects has also been reported [16]. However, assessment of nitric oxide concentrations has shown controversial results, with some researchers reporting a decrease in eNO during exercise in sedentary and active subjects, paralleled with an increased VO_2_ and ventilation. As yet, the behaviour of eNO some days after marathon has not been studied previously.

Another important finding in this study is the decrease in pulmonary function, as evidenced by FEV1, FVC, and PEF after marathon, which was sustained until 72 hours after marathon. In fact, it has been reported that respiratory muscles significantly fatigue during endurance activities such as the marathon, reducing maximal voluntary and expiratory pressures and peak inspiratory and expiratory flow rates [28].

The lungs have crucial role in gas exchange, experiencing great modifications of their activity during exercise, in addition to their important role in cardiopulmonary function [19]. Miles et al. [29] have reported a reduction in pulmonary diffusing capacity after prolonged exercise, which persisted for a certain period of time.

Finally, we found the so-called sport cardiac fatigue evidenced by a decrease in VO_2_ peak. In previous studies, we demonstrated a postmarathon drop in VO_2_ peak and O_2_ pulse, which is consistent with reduction in cardiac inotropism, but with pulmonary compensation, with greater ventilatory efficiency and optimization of O_2_ use to achieve the same exertion [2]. However, in this study, we also could observe a decrease in VO_2_ and VE peaks, bringing about an important cardiac fatigue without pulmonary compensation, which could possibly be explained by the increased pulmonary inflammation evidenced by an increase in inflammatory cells and eNO after marathon, with a decrease in cytokines, as explained above. Nevertheless, the important negative correlation between FEV1 and nitric oxide levels 24 and 72 hours after marathon and the positive correlation between FVC and VO_2_ peak after marathon suggest an adaptation of some athletes in reducing the bioavailability of eNO and maybe increasing pulmonary immunity to maintain cardiopulmonary capacity and reduce cardiac fatigue.

## 5. Conclusion

In summary, the present study shows an increase in nitric oxide in exhaled air and in inflammatory cell number, as well as a decrease in cytokines in sputum in marathoners after marathon running, which reflects an airway inflammation and impairment of the airway immune system, which in turn hinders cardiopulmonary capacity. However, those athletes who presented a lower eNO bioavailability presented a lower decrease in the cardiopulmonary capacity and in cardiac fatigue, highlighting the role of eNO in these parameters, as a plausible mechanism involved in this response.

## Figures and Tables

**Table 1:** Demographic characteristics and marathon time of marathoners (*n* = 31).

Variable	Mean±standard deviation
Age (years)	39 ± 9
Marathon time (minutes)	270 ± 40
Average speed (km/h)	9.5 ± 0.2
% of CPET peak speed	51.9 ± 0.1
Running volume (km)	57 ± 23

Data are shown in mean±standard deviation. Percentage of peak speed=average speed during marathon/peak speed in cardiopulmonary test ^∗^100, CPET: cardiopulmonary exercise test.

**Table 2:** Inflammatory cells and cytokines after marathon in sputum.

	Before	Immediately after
Total cells (×10^4^/mL)	143 ± 16	266^∗^ ± 43
Neutrophils (×10^4^/mL)	13 ± 10	61^∗^ ± 13
Macrophages (×10^4^/mL)	21 ± 6	54^∗^ ± 14
Epithelial cells (×10^4^/mL)	113 ± 12	201^∗^ ± 32
Lymphocytes (×10^4^/mL)	79 ± 221	53 ± 82
IL-6 (pg/mL)	581 ± 1529	87 ± 53
IL-8 (pg/mL)	3099 ± 6511	1450 ± 6233
IL-12p40 (pg/mL)	3775 ± 12406	285^∗^ ± 131
IL-23 (pg/mL)	3722 ± 12115	1004^∗^ ± 254
IL-33 (pg/mL)	412 ± 1546	267^∗^ ± 145
TSLP (pg/mL)	387 ± 1974	20^∗^ ± 16

Data are shown in mean±standard deviation. ^∗^*p* < 0.05. IL: interleukin.

**Table 3:** Pulmonary function before, immediately after, and 24 hours and 72 hours after marathon.

	24 h before	Immediately after	24 h after	72 h after
FVC (L)	4.9 ± 0.7	4.6 ± 0.7^∗^	4.8 ± 0.7^∗^	4.8 ± 0.7^∗^
FEV1 (L)	3.9 ± 0.6	3.8 ± 0.7^∗^	3.7 ± 0.7^∗^	3.8 ± 0.6^∗^
PEF (L/s)	10.07 ± 1.9	8.96 ± 2.1^∗^	9.17 ± 2^∗^	9.36 ± 1.9^∗^
FEV1/FVC	80.9 ± 5.5	81.5 ± 6.4	78.5 ± 9.3	78.8 ± 6.9^∗^

Data are shown in mean±standard deviation. FVC: forced vital capacity; FEV1: forced expiratory volume in one second; PEF: peak of expiratory flow. ^∗^*p* < 0.05 compared with basal values.

**Table 4:** Cardiopulmonary function behaviour before and after marathon.

Variable	Before marathon	After marathon	*p* value
HR at rest (bpm)	67 ± 9	65 ± 16	NS
Peak HR (bpm)	182 ± 13	181 ± 14	NS
Peak speed (km/h)	18 ± 2	18 ± 3	NS
Peak VO_2_ (mL·kg^−1^·min^−1^)	57 ± 6	55 ± 6	^∗^<0.01
Peak VE (L/min)	134 ± 19	132 ± 19	^∗^<0.05
O_2_ pulse (mL·btm)	22 ± 3	22 ± 3	NS
Peak PetO_2_	111 ± 6	114 ± 4	^∗^<0.05
VE/VCO_2_ slope	26 ± 3	26 ± 2	NS

Data are shown in mean±standard deviation. HR: heart rate; VO_2_: oxygen consumption; VE: ventilation; O_2_: oxygen; PetO_2_: end-tidal oxygen tension.

## Data Availability

The data used to support the findings of this study are available from the corresponding author upon request.
